# Variations in Routine Childhood Vaccination Gaps: A Decomposition Analysis Across 80 Low- and Middle-Income Countries

**DOI:** 10.3390/vaccines13111136

**Published:** 2025-11-04

**Authors:** David Phillips, Jordan-Tate Thomas, Gloria Ikilezi

**Affiliations:** Gates Foundation, Exemplars in Global Health Program, Seattle, WA 98109, USA; jordan-tate.thomas@gatesfoundation.org (J.-T.T.); gloria.ikilezi@gatesfoundation.org (G.I.)

**Keywords:** routine immunization, DTP3 coverage, zero dose, missed DTP, drop-out, Immunization Agenda 2030 (IA2030), decomposition analysis, contributing factors

## Abstract

**Background**: Despite remarkable progress in expanding access to childhood vaccines in the last two decades, global coverage with the third dose of the diphtheria–tetanus–pertussis-containing vaccine (DTP3) has recently plateaued, with many countries yet to meet the targets of the Immunization Agenda 2030 (IA2030). As countries cluster around the 80% coverage mark, further gains require targeted interventions for unreached populations. This analysis disaggregates children missing DTP3 into three groups—zero dose (ZD), missed DTP (MD), and drop-out (DO)—which, with DTP3, form four mutually exclusive groups, and examines which of these groups contributes most to coverage changes across countries. **Methods**: A total of 295 Demographic and Health Surveys from 1986 to 2023 were analyzed across 80 countries, comprising over 2.4 million children. Children were classified into mutually exclusive groups: DTP3, ZD, MD, and DO. We described trends over time and conducted decomposition analyses using a naïve approach and a structural model with isometric log-ratio transformations and causal mediation pathways. **Results**: Among the 2.4 million children across 80 countries, 63.8% had received DTP3, while 16.2% were DO, 8.8% were MD, and 11.2% were ZD. Countries showed important variations: some mainly reduced ZD, others reduced MD or DO, many achieved balanced progress, and a few experienced setbacks. The naïve model showed that coverage changes reflected different combinations of shifts across ZD, MD, and DO depending on context. The structural model indicated that DO had the strongest direct association with DTP3 coverage, followed by MD and ZD. **Conclusions**: This analysis highlights the differential contribution of intermediate groups to coverage variations over time. Understanding the association between coverage gains and shifts in ZD, MD, or DO can complement existing strategies to inform targeted planning and accelerate progress towards IA2030 equity goals.

## 1. Introduction

The global scale-up of routine childhood immunization is among the most successful global health interventions in history, estimated to have saved tens of millions of lives over the past five decades [1,2]. To sustain this impact, countries around the world have made steady investments to improve immunization coverage and expand equitable access to vaccines [1,2].

More recently, global efforts such as the Global Vaccine Action Plan (GVAP), which ran from 2011 to 2020, were endorsed by countries worldwide to promote a shared commitment toward expanding access to vaccines and preventing millions of deaths [3,4]. Following GVAP, the Immunization Agenda 2030 (IA2030) was launched in 2021 and endorsed by over 140 national governments and global partners [5]. IA2030 aims to recover and strengthen immunization systems, particularly in light of disruptions caused by the COVID-19 pandemic. Although some countries have made progress, many have not yet returned to pre-pandemic coverage levels, let alone continued the trends of the GVAP era [6,7]. According to a recent IA2030 report, the world is not currently on track to meet most of the immunization targets for 2030 [7]. Global coverage of the third dose of the diphtheria, tetanus, and pertussis vaccine (DTP3), a widely used marker of routine immunization performance, stagnated at 84 percent in 2023, slightly down from 86 percent in 2019 [6,7,8,9].

This plateauing in global coverage in recent years suggests that current strategies in many countries may be insufficient—not only to reach remaining unvaccinated and under-immunized populations, but also to ensure that each new birth cohort is consistently connected with routine immunization [6]. In several countries that have approached or surpassed 80 percent coverage, further improvements have proven especially difficult—an experience sometimes described as an immunization “glass ceiling” [10].

Although the phenomenon of plateauing immunization coverage appears to be a global trend, the notion of a “glass ceiling” should not suggest that all countries face the same constraints or require the same solutions. Countries may have taken different paths to reach their current levels of coverage and may need to pursue different approaches to advance further [9,11]. To close remaining gaps and break through the ceiling, it may be essential to tailor strategies to each country’s unique vaccination barriers and opportunities for improvement [11].

Some of the world’s most successful countries in terms of vaccine delivery have done exactly that. Initiatives such as Exemplars in Global Health have highlighted how countries have successfully reached underserved populations and closed equity gaps by employing context-specific strategies that build on what is needed locally [12,13].

While success stories about improving immunization coverage offer useful lessons, translating them into sustained gains elsewhere remains a challenge. Countries exhibit different patterns of which children are missed, and these patterns are not always well distinguished in existing monitoring approaches. Supply- and demand-side drivers of coverage, while important, are difficult to define and measure consistently between countries [14,15,16]. Combined with limitations in available data, this has made it difficult to identify which factors have contributed most to changes in coverage and which delivery strategies from one context may be appropriate in another [2,4]. An approach that instead focuses on disaggregating coverage into easily measured intermediate outcomes can provide a structured way to describe where progress has occurred and where gaps remain, complementing existing tools for understanding immunization gaps [14,17].

Previous studies have identified these intermediate outcomes individually. Cata-Preta et al. (2021) [14] described the immunization cascade, quantifying drop-out at each stage, i.e., children who started DTP but did not complete the series [18]. Wonodi et al. (2023) analyzed cross-antigen relationships, examining characteristics of children vaccinated against some diseases but not others compared with those never vaccinated [17]. Together, these studies define the complete set of groups in relation to the DTP3 gap—children who have never been vaccinated; those who have received some vaccines such as BCG, polio, or MCV, but not DTP; and those who have failed to complete their DTP regimen. In this analysis, we explicitly define these three groups to form a mutually exclusive and collectively exhaustive set of intermediate outcomes that decompose and represent the decomposition components of non-DTP3 coverage, ensuring that all children are accounted for without overlap or omission. The full model comprises four groups in total (DTP3, ZD, MD, and DO).

Building on this classification, we introduce a model for analyzing vaccine utilization through a decomposition of changes in routine immunization coverage. Specifically, we apply the ZD, MD, and DO categories to examine country-by-country changes in DTP3 coverage and to identify which groups have driven past gains or setbacks. Using readily available secondary data, this approach provides a standardized way to describe what changes in coverage may be linked to. While not a replacement for multi-antigen coverage analyses, it complements existing tools by enabling consistent decomposition across countries and over time and supporting programmatic discussions on where additional efforts may be most needed.

## 2. Methods

### 2.1. Conceptual Model

This analysis built on previous work to define three groups that represent the differences between observed DTP3 coverage and 100% DTP3 coverage [14]:

Zero dose (ZD)—the proportion of eligible children who received no doses of the following vaccines: Bacille Calmete–Guérin (BCG), any vaccines against poliomyelitis (polio), measles-containing vaccine (MCV), or DTP-containing vaccines (pentavalent, DTAP, TDAP, or DTP + Hib).

Missed DTP (MD)—the proportion of eligible children who received at least one dose of BCG, polio, or MCV, but no dose of DTP.

Drop-out (DO)—the proportion of children who received at least one dose of DTP (DTP1) but not three (DTP3).

These components are mutually exclusive (e.g., a child cannot be both ZD and MD simultaneously), and they are collectively exhaustive (i.e., all children must be in one of the four groups, including DTP3 coverage). Accordingly, the analysis treats ZD, MD, and DO as the decomposition components of non-DTP3 coverage, and the full model comprises four mutually exclusive groups in total. These are desirable properties for decomposition analysis, since they mean that the groups form complete compositional data. In other words, within a given eligible population, any change in DTP3 coverage must be offset by a change in one or more of the other categories. While broader demographic shifts may also influence coverage levels, this decomposition isolates the immunization-related pathways [15].

### 2.2. Data and Variable Definitions

We compiled 295 publicly available Demographic and Health Surveys (DHSs) with children’s health modules from 80 countries conducted between 1986 and 2023 into a single dataset [18]. Data were downloaded on February 26, 2025. All data were extracted at the individual child level. Each DHS is a nationally representative household survey implemented by country governments in collaboration with the DHS Program. Data collection procedures are standardized across countries, with comprehensive methodological documentation available through the DHS Program [18].

We defined a child as ZD if they were recorded as “no” for BCG, polio1, MCV1, and DTP1. We defined a child as MD if they were recorded as vaccinated with at least one of BCG, polio, or MCV, but not DTP. We defined a child as DO if they were recorded as vaccinated with one dose of DTP but not three. All definitions accepted vaccination status according to a vaccination card or as reported by the survey respondent, but sensitivity analysis explored the difference if using a vaccination card only. Children recorded as “Don’t know”, “Not in universe”, or with missing values for BCG, polio1, MCV1, DTP1, or DTP3 were excluded from the analysis. Children recorded as not vaccinated for DTP1 but vaccinated for DTP3 were also excluded from the analysis.

### 2.3. Analysis

We conducted descriptive analysis and decomposition analysis at the national level. The descriptive analysis visualized the share of non-DTP3 children in each survey falling into the three remaining groups (ZD, MD, and DO), excluding those with DTP3 coverage. We visualized these proportions over time, aggregated to the above geographic levels.

Decomposition analysis took two approaches: a “naïve” model and a “structural” model. Both approaches analyzed the contribution of each of the three decomposition components (ZD, MD, and DO) toward overall DTP3 change between 2000 and 2020 (or the nearest years with available data), excluding survey time points between those years or countries with only a single data point.

The naïve model simply described the proportion of overall change in DTP3 coverage attributable to shifts in each of the three decomposition components, as shown in Formula (1):

Formula (1). Naïve model for decomposing change in DTP3 coverage explained by change in DO, MD, or ZD (only DO is shown):

(1) $$Contribution_{DO}=\;\frac{\left|DO_{2020}-DO_{2000}\right|}{\left|DO_{2020}-DO_{2000}\right|+\left|MD_{2020}-MD_{2000}\right|+\left|ZD_{2020}-ZD_{2000}\right|}$$

(ZD: zero-dose prevalence, MD: missed-DTP prevalence, DO: DTP drop-out prevalence.)

**Figure 1:**
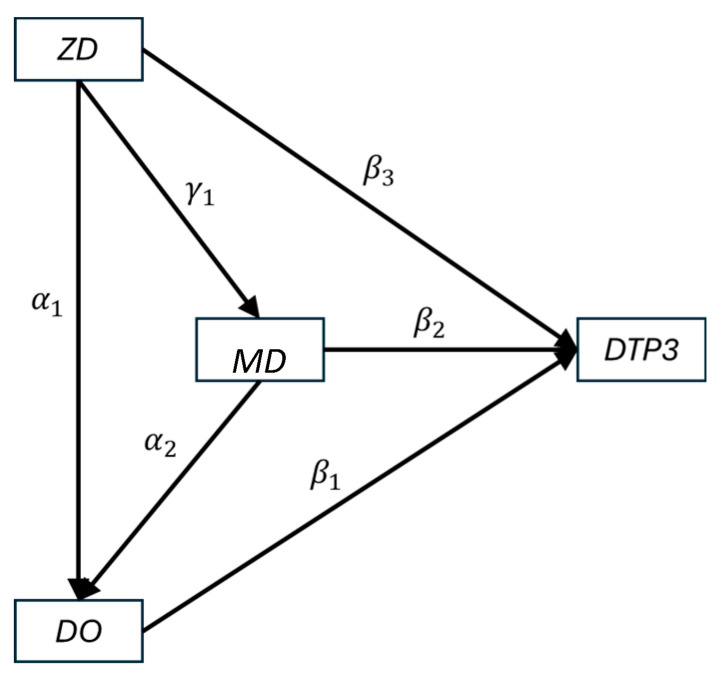
Causal relationships represented in structural model. ZD: zero-dose prevalence, MD: missed-DTP prevalence, DO: DTP drop-out prevalence, DTP3: DTP3 coverage.

The structural model used a regression-based approach to account for the relationships between ZD, MD, DO, and DTP3, namely, causal mediation. In this model, variation in ZD may be associated with variation in MD, DO, or DTP3; variation in MD may be associated with DO or DTP3; and variation in DO is directly associated with DTP3. This ordering reflects the logical sequence of the vaccination pathway shown in Figure 1, but the model does not assume that all changes are improvements: increases or decreases in one category can be associated with corresponding increases or decreases in others. We used the lavaan package (version 0.6-20) in R version 4.5.1 (R Foundation for Statistical Computing, Vienna, Austria) to estimate these pathways as a single model, capturing direct, indirect, and total effects [16]. The associated model code is provided in Appendix A [18].

The structural model also accounted for the compositional nature of the data, meaning that the four variables in the analysis must sum to 100%. We applied an isometric log-ratio (ILR) transformation to eliminate the constant-sum constraint and transform the variables into Euclidean space, allowing the use of standard statistical methods [16,17,18]. We conducted a sensitivity analysis comparing the ILR transformation with an alternative transformation (additive log-ratio, ALR), which is presented in Appendix A [16,17,18]. The formula used for sequential ILR transformations was as follows:

Formula (2). Isometric log ratio transformations:

(2) $$z_{1}=\sqrt{\frac{1}{2}}\ln\frac{DO}{DTP3}$$

$$z_{2}=\sqrt{\frac{2}{3}}\ln\frac{MD}{{\left(DTP3*DO\right)}^{\frac{1}{2}}}$$

$$z_{3}=\sqrt{\frac{3}{4}}\ln\frac{ZD}{{\left(DTP3*DO*MD\right)}^{1/3}}$$

(ZD: zero-dose prevalence, MD: missed-DTP prevalence, DO: DTP drop-out prevalence, DTP3: DTP3 coverage [17,18].)

For interpretability, the results herein are presented using the original variable labels (ZD, MD, and DO) rather than their ILR labels (z1, z2, and z3).

## 3. Results

### 3.1. Data

We analyzed 295 publicly available demographic health surveys from 80 countries spanning 1986 to 2023. This combined dataset included a sample of 2,447,247 children who were eligible for the relevant vaccines at the time of their survey. Overall, 63.8% of children in the sample were reported as having at least three doses of a DTP-containing vaccine, 16.2% were counted as drop-out (DO), 8.8% were counted as missed DTP (MD), and 11.2% were counted as zero dose (ZD).

### 3.2. Descriptive Analysis

**Figure 2:**
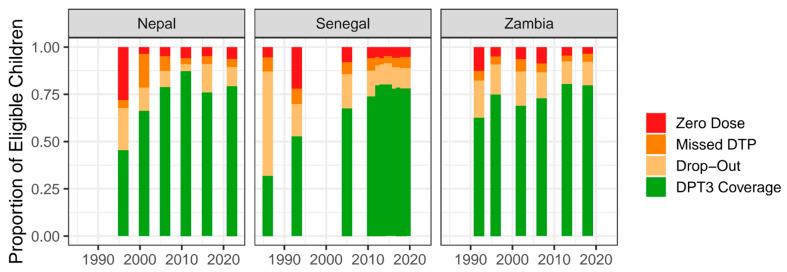
Descriptive analysis of DTP3 coverage, drop-out prevalence, under-immunization prevalence, and zero-dose prevalence for three countries studied as exemplars in vaccine delivery: Nepal, Senegal, and Zambia. 1. Zero dose (ZD)—the proportion of eligible children who received no doses of the following vaccines: Bacille Calmete-Guérin (BCG), any vaccines against poliomyelitis (polio), measles-containing vaccine (MCV), or DTP-containing vaccines (pentavalent, DTAP, TDAP, or DTP + Hib). 2. Missed DTP (MD)—the proportion of eligible children who received at least one dose of BCG, polio, or MCV, but no doses of DTP. 3. Drop-out (DO)—the proportion of children who received at least one dose of DTP (DTP1) but not three (DTP3).

Figure 2 demonstrates the results of the descriptive analysis for the three Vaccine Delivery Exemplar countries: Nepal, Senegal, and Zambia. Each bar of the figure represents all eligible children, broken down into the share of children who were recorded as DTP3, DO, MD, or ZD. As can be seen in the figure, the composition of gaps at baseline differed sharply between countries, shaping how progress was achieved over the following two decades. In Nepal in 1996, nearly one-third of children were zero dose (28.0%), compared to much smaller shares in MD (17.6%) and DO (8.4%), resulting in DTP3 coverage of 45.5%. In contrast, Senegal in 1986 had a relatively small ZD population (6.0%) but a very large DO group (43.6%), with DTP3 coverage of 31.9%. These distinct starting points meant that each country’s gains came through different pathways: Nepal’s coverage rose to 79.3% by 2022 through large reductions in ZD (28.0% to 3.7% between 1996 and 2001), followed by declines in MD (17.6% to 7.8% between 2001 and 2006) and DO (8.4% to 3.7% between 2006 and 2011). Senegal’s coverage increased to 78.1% by 2019, driven primarily by reductions in DO, while ZD remained relatively low throughout. Zambia offers a third example, with DTP3 coverage increasing from 62.6% in 1992 to 79.8% in 2018, and progress was achieved through more balanced reductions across ZD, MD, and DO. Together, these examples highlight how very different baseline realities required different routes to progress. Supplementary Figure S1 displays these figures for every country included in the analysis.

**Figure 3:**
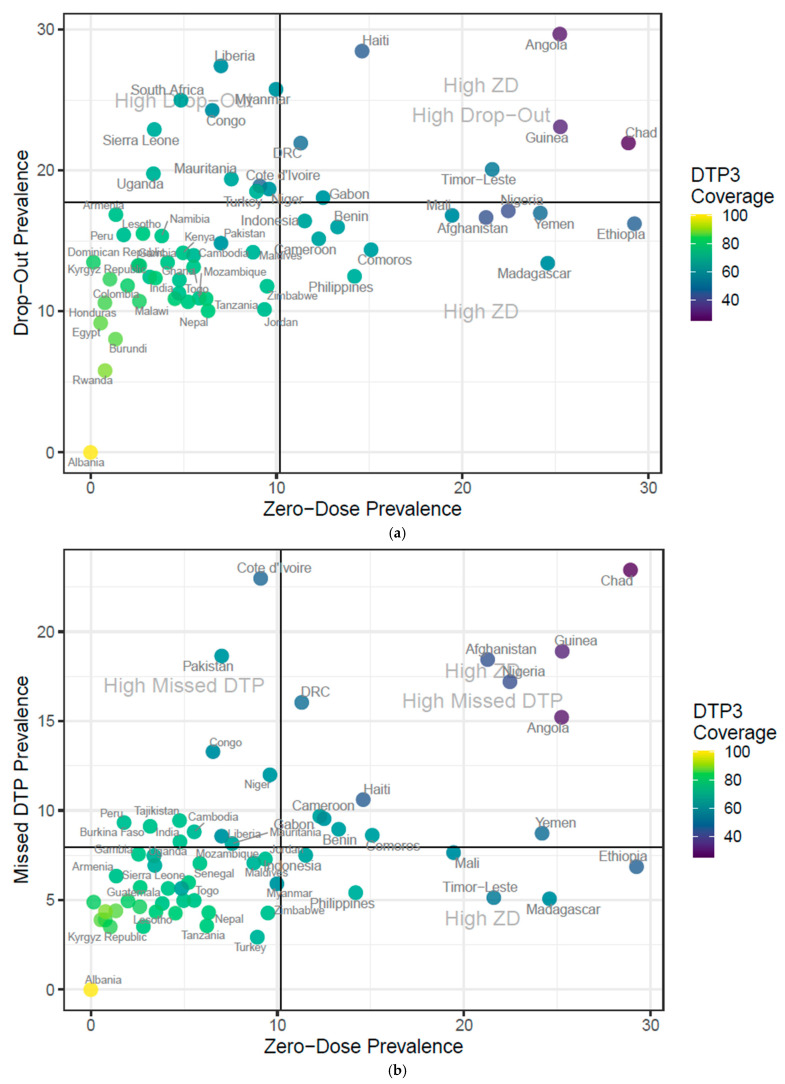
(**a**) Zero-dose prevalence compared to drop-out prevalence, all available countries. (**b**) Zero-dose prevalence compared to missed-DTP prevalence, all available countries. 1. Zero-dose prevalence (ZD)—the proportion of eligible children who received no doses of the following vaccines: Bacille Calmete–Guérin (BCG), any vaccines against poliomyelitis (polio), measles-containing vaccine (MCV), or DTP-containing vaccines (pentavalent, DTAP, TDAP, or DTP + Hib). 2. Drop-out prevalence (DO)—the proportion of children who received at least one dose of DTP (DTP1) but not three (DTP3). 3. Missed-DTP prevalence (MD)—the proportion of eligible children who received at least one dose of BCG, polio, or MCV, but no doses of DTP.

**Figure 4:**
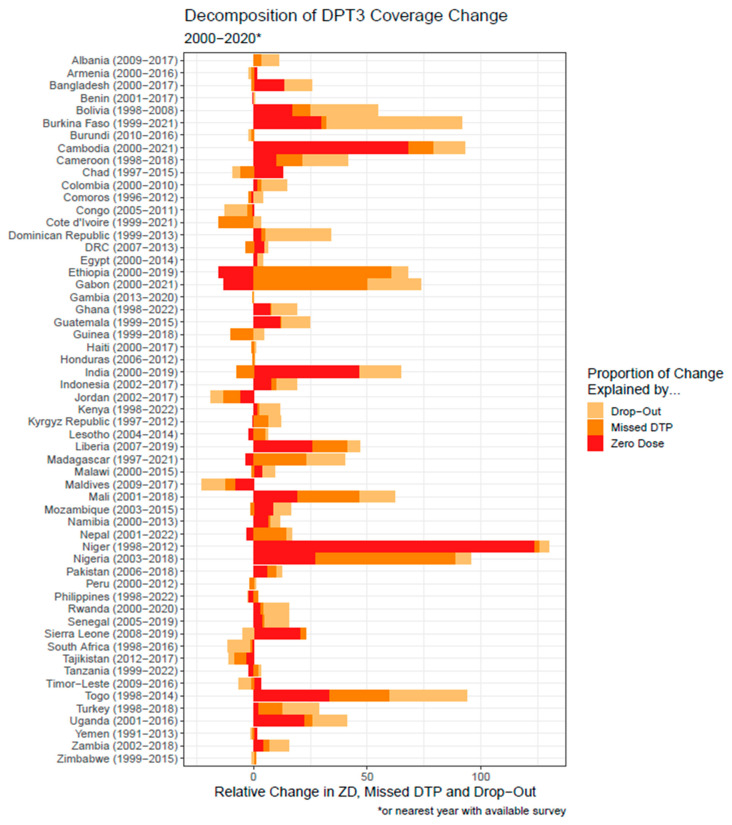
Changes in zero-dose, missed-DTP, and drop-out prevalence by country for 57 countries with available data in two time points. 1. Zero dose (ZD)—the proportion of eligible children who received no doses of the following vaccines: Bacille Calmete-Guérin (BCG), any vaccines against poliomyelitis (polio), measles-containing vaccine (MCV), or DTP-containing vaccines (pentavalent, DTAP, TDAP, or DTP + Hib). 2. Missed DTP (MD)—the proportion of eligible children who received at least one dose of BCG, polio, or MCV, but no doses of DTP. 3. Drop-out (DO)—the proportion of children who received at least one dose of DTP (DTP1) but not three (DTP3).

Figure 3a,b display these percentages across all countries included in the analysis for the most recent year, with data available since 2010. Figure 3a shows zero-dose prevalence compared to drop-out prevalence, highlighting countries that have above-average percentages in one group or both. Figure 3b displays zero-dose prevalence compared to missed-DTP (MD) prevalence in a similar manner. Figure 3a demonstrates that Liberia, Myanmar, South Africa, DRC, Republic of Congo, Uganda, and Niger have notably high DO prevalence (above the average DO across countries in the figure). Figure 4 demonstrates that Republic of Congo, Pakistan, Cote d’Ivoire, and Liberia have notably high MD prevalence. Both figures also demonstrate that Ethiopia, Yemen, Nigeria, Afghanistan, Mali, Madagascar, Benin, and Cameroon have a notably high share of ZD.

### 3.3. Decomposition Analysis

Figure 4 displays results from the naïve model. In this figure, the length of each bar represents the overall change in DTP3 coverage from 2000 to 2020 (or the nearest available year), with positive values representing percentage-point increases in DTP3 coverage and negative values representing decreases. The colored portions of the bars reflect the proportions of this overall change attributable to shifts in ZD, MD, and DO, as defined in Formula (1). From this figure, it is apparent that some countries, such as Niger, made nearly all of their progress by reducing ZD, while others, such as Burkina Faso, achieved gains primarily through reductions in DO. Many countries, including India and Mali, show relatively balanced improvements across all groups. The figure also highlights that in some countries, progress in one group occurred alongside setbacks in another. Ethiopia is one example: overall DTP3 coverage increased between 2000 and 2020, even though the ZD category grew, because reductions in MD and DO were large enough to offset that increase. A small number of countries, such as South Africa, experienced net declines in DTP3 coverage, where growth in the ZD and DO categories outweighed reductions in MD.

Table 1 displays results from the structural model. The first three parameters, $\beta_{1},\;\beta_{2}$, and $\beta_{3}$, represent the direct effects of DO, MD, and ZD on DTP3 coverage. $\beta_{1}$ has a value or −0.243 (standard error: 0.008, *p*-value < 0.001) indicating that a reduction in the isometric log-ratio of DO has a larger direct effect on DTP3 than either MD (−0.066, standard error: 0.007, *p*-value < 0.001) or ZD (−0.039, standard error: 0.003, *p*-value < 0.001). The parameters $\alpha_{1}$, $\alpha_{2}$, and $\gamma_{1}$ represent the indirect effects of ZD on DO, MD on DO, and ZD on MD, respectively. Accounting for mediation using these coefficients, the indirect effect of ZD on DTP3 via DO is −0.022 (standard error: 0.006, *p*-value < 0.001), the indirect effect of ZD on DTP3 via MD is −0.005 (standard error: 0.002, *p*-value = 0.014), and the indirect effect of MD on DTP3 via DO is −0.108 (standard error: 0.012, *p*-value < 0.001). Taken together, the total effect of ZD on DTP3 is calculated to be −0.067 (standard error: 0.007, *p*-value < −0.001), the total effect of MD on DTP3 is calculated to be −0.174 (standard error: 0.012, *p*-value < 0.001), and the total effect of DO on DTP3 ($\beta_{1}$) is −0.243 (standard error: 0.008, *p*-value < 0.001). This can be interpreted as a reduction in DO having the largest total effect on DTP3, followed by MD and ZD.

## 4. Discussion

Despite widespread investments in routine immunization, many countries have reached a plateau in DTP3 coverage, with progress slowing or stalling in recent years [2,6]. This phenomenon, countries stalling close to an 80% coverage level, can be described as an immunization “glass ceiling,” a concept highlighted in recent global discussions emphasizing the need for innovations to overcome persistent coverage barriers [10]. Aggregate coverage metrics alone offer limited insights into why gains have stalled or how remaining gaps can be closed [8,9,14]. This analysis disaggregates the population of children not fully vaccinated with DTP3 into three mutually exclusive categories: zero dose, missed DTP, and drop-out. By isolating these groups, we aimed to better characterize where children are being lost along the pathway to DTP3 coverage and to reveal patterns suggestive of country-level priorities, especially those tied to persistent inequities in access and utilization of immunization services among harder-to-reach populations [14,19,20].

This approach allows for a more precise interpretation of the remaining DTP3 gap in each country, offering a standardized lens to distinguish between different sources of missed vaccinations. By separating zero-dose, missed-DTP, and drop-out children, countries can better understand which broad areas of intervention may be most relevant to their context. By distinguishing groups that require different types of outreach and follow-up, countries can better assess which specific barriers are most limiting progress. For example, a high prevalence of missed DTP may call for different interventions than those needed to reach zero-dose children or reduce drop-out. These distinctions are especially important given the compositional nature of immunization coverage: for the purposes of this analysis, all children must fall into one of these categories, meaning any increase in DTP3 coverage must be reflected as a reduction in one or more of the other categories. Recognizing this dynamic highlights the pathways through which coverage can improve and provides a foundation for future analyses, including subnational approaches, that more directly address within- and between-country inequalities in immunization coverage and vaccine access [5,7].

One interpretation of this descriptive analysis is that zero dose, missed DTP, and drop-out represent different programmatic challenges in different settings, depending on local prevalence. While global estimates often highlight zero dose as the largest group, multi-country and subnational analyses consistently reveal substantial variation, with no single category predominant across all contexts [21,22,23]. To quantify the relative contribution of each group, we estimated a structural model across all countries combined; these results provide a summary of broad relationships among ZD, MD, DO, and DTP3. However, the descriptive results make clear that country-specific pathways differ substantially, and the pooled model should not be interpreted as evidence of a single uniform pathway applying in all settings. Together, these findings complement the broader body of information available to programs, helping global, regional, and country stakeholders frame discussions about where additional attention may be most warranted. Although the nature of this paper is primarily analytical, its findings contribute to epidemiological understanding by characterizing population-level patterns of vaccine uptake and identifying where programmatic challenges occur. In doing so, this analysis supports broader public health and policy efforts to improve immunization coverage and equity.

To our knowledge, this analysis is the first to bring together two related perspectives in immunization monitoring. The first is the widely used framing of zero dose in contrast to drop-out, which typically considers DTP0 as a proxy indicator for zero-dose prevalence. The second is a growing perspective that DTP0 actually comprises two distinct population groups: children who have truly never been vaccinated and children who have been vaccinated but not with a DTP-containing vaccine. The analysis by Wonodi 2023 points out that the latter population (termed “misclassified zero dose” in that study) has an empirically different vulnerability profile than truly zero-dose children [24]; they tend to be less rural, higher-income, and utilize other health services more frequently, among other characteristics. We term this group “missed DTP”, including children who received some vaccine (BCG, polio, or MCV) but not DTP. We agree with the argument that it is meaningful to distinguish this population group, as it represents children who are reachable through campaigns and birth doses, but were not reached through routine immunization. Similarly, the distinction between the drop-out population and the zero-dose population has been shown to stem from distinctly different socioeconomic circumstances, health systems, and other determinants, which can be encapsulated with existing frameworks [14,15]. For example, one study in Kwara, Nigeria, highlighted the strong association between age, home birth, and stock-outs in distinguishing between zero dose and drop-out, among other factors [25]. Recognizing all three mutually exclusive groups thus allows for a more granular interpretation of coverage gaps and clearer identification of country- and subnational-level priorities. It also highlights that the share of children classified as zero dose is generally lower than reported elsewhere when other antigens are considered [14,22,23,26,27,28].

The strategies required to bring these population groups up to three doses of DTP are therefore likely to differ. Studies about closing the drop-out gap have highlighted reminder systems, incentives, and patient experience as effective strategies to keep children in the routine immunization system provided that they have already begun using it [29]. Conversely, vaccinating the missed-DTP population with DTP depends on strategies to link reached populations to their nearest routine immunization site in the case of campaigns and supplementary immunization activities, or to encourage and facilitate the return of mothers and caregivers of children who received a birth dose [30,31]. Finally, zero-dose populations may require more locally designed and tailored strategies to address the intersecting underlying determinants that impede intent to vaccinate, access to available vaccination services, optimal facility readiness, and service delivery.

An important consideration is that Figure 3 and Figure 4 display the (absolute) prevalence of zero dose, missed DTP, and drop-out, not the (relative) share each contributes to the overall DTP3 coverage gap. As such, countries with larger overall coverage gaps fall into the highlighted quadrants, while countries with high DTP3 coverage but remaining priorities are more difficult to discern. This is not intended to imply that prioritizing between the three factors is no longer necessary for high-coverage countries; rather, every country has a distinct set of challenges, even if DTP3 coverage is relatively high.

The naïve model presented here presents a general understanding of which group changed the most during the time period, but it likely overstates the extent to which a reduction in zero-dose prevalence leads to a direct increase in DTP3 coverage. In the structural model, much of the effect of reducing zero-dose prevalence is mediated through changes in missed DTP and drop-out. In other words, the benefit of reducing zero dose is largely indirect, while the strongest direct effect on DTP3 coverage comes from reducing drop-out prevalence. To our knowledge, this is the first analysis to explicitly acknowledge and quantify this mediation pathway.

Several other studies have analyzed the correlation between zero-dose prevalence and DTP3 coverage, generally treating declines in zero dose as direct contributions to improved coverage. However, these approaches have not always distinguished whether reductions in zero dose were accompanied by changes in drop-out or missed DTP [22,23,26,27]. While prior research has examined factors contributing to changes in coverage, to our knowledge no study has applied a decomposition framework of this type to disentangle the relative contributions of zero dose, missed-DTP, and drop-out [23,32].

The structural model presented here is designed to approximate causal pathways, given the deterministic, compositional nature of the variables and the fact that all key relationships are explicitly represented. Nonetheless, broader dynamics—such as changes in mortality distributions (e.g., during pandemics); population movements through migration or displacement; and underreporting in household surveys that systematically exclude certain groups, such as mobile or displaced populations—may also influence observed coverage changes and are not directly captured in this analysis. A more robust study design could be to use longitudinal data and decompose changes at the individual level, but individual linkage is not possible with the data used in this analysis. By incorporating 80 countries with comparable data spanning over three decades, this study design has its own advantage over individual-level analysis; however, very few countries have such longitudinal data.

The structural model itself may be an advancement in the field, as compositional data in general, let alone causal mediation analysis with compositional data, are unusual in public health settings. One known study deployed a similar approach to decompose maternal mortality trends into components of fertility and survival [33]. A separate study analyzed compositional nutrition data using the same isometric log-ratio transformation presented here [34]. However, neither analysis modeled the complete causal diagram using both compositional analysis techniques and SEM that captures mediation. One of the few studies that did so is known as the MINISTOP study, which applied very similar methods (SEM on ILR-transformed variables) to physical activity data [35]. Use of the term “decomposition analysis” subtly varies within the literature as well; at times it is used in reference to separating direct and indirect effects, and at other times it is used in reference to data that are compositional [29,36]. We are unaware of any published study in the literature that has conducted this kind of causal mediation analysis on compositional data on immunization.

However, this analysis has several limitations. Data from demographic health surveys contain well-documented recall, non-response, survival, and other biases that may skew estimates of DTP3 coverage and the prevalence of zero dose, missed DTP, and drop-out [37,38,39,40,41]. Evaluating the direction of these biases and their impact on the effect sizes and priorities we present was beyond the scope of this study, but previous analyses suggest that recall bias tends to over-estimate drop-out. Future research may assess corrections to these biases. Furthermore, although we argue that there are many advantages to analyzing these indicators of non-vaccination, they do not offer insights into the underlying causes of zero-dose, missed-DTP or drop-out behavior. Mixed-methods analysis of the root causes of each factor in local contexts may be warranted to gain a deeper explanation of why children remain unvaccinated. Finally, this analysis does not recommend interventions to improve vaccination; it merely helps define intermediate outcomes that interventions may aim to effect on the causal pathway to full coverage. We also acknowledge that while this analysis spans the years 1986–2023, the post-pandemic period is of particular interest for future work, as this framework could help quantify and interpret coverage recovery following COVID-19-related disruptions.

The results and discussion in this manuscript may be interpreted as potentially shifting the focus from the importance of reaching zero dose children for immunization. While this analysis may point to a lower prevalence and smaller direct effect sizes than previously reported, the well-documented inequities that persist among zero-dose populations must be acknowledged as a separate justification for addressing zero-dose prevalence beyond vaccine coverage alone.

Focusing on DTP3 coverage, this analysis has two primary implications. First, the prevalence of zero dose, missed DTP, and drop-out vary markedly across settings, highlighting the importance of tailoring interventions to local barriers. Second, we show that reductions in drop-out have historically had a stronger effect on DTP3 coverage than reductions in missed DTP, and that reductions in missed DTP have had a stronger effect than reductions in zero dose. Consistent with these descriptive patterns, findings from the structural model suggested stronger direct effects on DTP3 from addressing DO relative to MD and ZD. Taken together, this means that countries with a high drop-out prevalence may need to consider placing greater emphasis on retaining children in the vaccination series. By contrast, those with higher missed-DTP or zero-dose prevalence face more nuanced trade-offs: reducing the prevalence of these groups alone may simply shift children into a different group without fully increasing DTP3 coverage.

## 5. Conclusions

In order to improve coverage and reduce vaccine inequalities within and between countries, program priorities must simultaneously consider the prevalence of the barriers contributing to their local “glass ceilings” and the effect that reducing each barrier would be expected to have. This analysis could be leveraged as a useful approach by policymakers, funders, and program implementers to identify what has driven vaccine coverage change most prominently between zero-dose, missed-DTP, and drop-out reductions, and what remains a priority for intervention, thereby facilitating both targeted planning and lesson sharing of best practices between geographies. Newer and more precise interpretations of existing data may be critical to address these inequities and deliver higher coverage of life-saving vaccines worldwide.

## Figures and Tables

**Table 1:** Structural model results: regression coefficients, variances, indirect effects, and total effects.

Parameter	Outcome (Y)	Explanatory (X)	Estimate	Standard Error	*p*-Value
Regressions					
$\beta_{1}$	DTP3	DO	−0.243	0.008	0.000
$\beta_{2}$	DTP3	MD	−0.066	0.007	0.000
$\beta_{3}$	DTP3	ZD	−0.039	0.003	0.000
$\alpha_{1}$	DO	ZD	0.092	0.023	0.000
$\alpha_{2}$	DO	MD	0.443	0.045	0.000
$\gamma_{1}$	MD	ZD	0.076	0.030	0.011
Variances					
	DTP3		0.003	0.000	0.000
	DO		0.164	0.013	0.000
	MD		0.274	0.023	0.000
Indirect Effects					
$\alpha_{1}\times \beta_{1}$	DTP3 (indirect effect via DO)	ZD	−0.022	0.006	0.000
$\gamma_{1}\times \beta_{2}$	DTP3 (indirect effect via MD)	ZD	−0.005	0.002	0.014
$(\alpha_{1}\times \beta_{1})+\left(\gamma_{1}\times \beta_{2}\right)$	DTP3 (total indirect effect)	ZD	−0.027	0.006	0.000
$\alpha_{2}\times \beta_{1}$	DTP3 (total indirect effect)	MD	−0.108	0.012	0.000
Total Effects					
$(\alpha_{1}\times \beta_{1})+\left(\gamma_{1}\times \beta_{2}\right)+\beta_{3}\;$	DTP3 (total effect)	ZD	−0.067	0.007	0.000
$\left(\alpha_{2}\times \beta_{1}\right)+\beta_{2}\;$	DTP3 (total effect)	MD	−0.174	0.012	0.000
$\beta_{1}$	DTP3	DO	−0.243	0.008	0.000

1. Zero dose (ZD)—the proportion of eligible children who received no doses of the following vaccines: Bacille Calmete–Guérin (BCG), any vaccines against poliomyelitis (polio), measles-containing vaccine (MCV), or DTP-containing vaccines (pentavalent, DTAP, TDAP, or DTP + Hib). 2. Missed DTP (MD)—the proportion of eligible children who received at least one dose of BCG, polio, or MCV, but no doses of DTP. 3. Drop-out (DO)—the proportion of children who received at least one dose of DTP (DTP1) but not three (DTP3).

## Data Availability

The data used in this study are publicly available from the Demographic and Health Surveys (DHS) Program at https://dhsprogram.com. Access requires free registration.

## Supplementary Materials

The following supporting information can be downloaded at https://www.mdpi.com/article/10.3390/vaccines13111136/s1, Supplementary Figure S1: Country-level descriptive decomposition graphs for all 80 countries are available in the Supplementary Figures.

## Abbreviations

ALR	Additive log-ratio
BCG	Bacille Calmette–Guérin vaccine
COVID-19	Coronavirus disease 2019
DHS	Demographic and Health Surveys
DO	Drop-out (received at least DTP1 but not DTP3)
DTP	Diphtheria–Tetanus–Pertussis–containing vaccine
DTP0/DTP1/DTP3	0/1/3 doses of DTP-containing vaccine
GVAP	Global Vaccine Action Plan
IA2030	Immunization Agenda 2030
ILR	Isometric log-ratio
MCV	Measles-containing vaccine
MD	Missed DTP (received ≥1 non-DTP vaccine, such as BCG, polio, or MCV, but no DTP)
SEM	Structural Equation Modeling
UNICEF	United Nations Children’s Fund
WHO	World Health Organization
ZD	Zero dose (no recorded doses of BCG, polio, MCV, or DTP)

## Appendix A

Model code:
model = ‘
# define direct effects
dpt3_cvg ~ b1 * dropout_prevalence + b2 * misseddtp3_prevalence + b3 * zd_prevalence

# define mediator effects
dropout_prevalence ~ a1 * zd_prevalence
dropout_prevalence ~ c1 * misseddtp3_prevalence
misseddtp3_prevalence ~ a2 * zd_prevalence

# define indirect and total effects of ZD
zd_indirect_DO := a1 * b1
zd_indirect_MD := a2 * b2
zd_total_indirect := zd_indirect_DO + zd_indirect_MD
zd_total_effect := b3 + zd_total_indirect

# define indirect and total effects of MD
md_total_indirect := c1 * b1
md_total_effect := b2 + md_total_indirect
’

Comparison of isometric log-ratio model and an alternative model (additive log-ratio):

Appendix A Table A1 displays a comparison of the isometric log-ratio (ILR) transformation used for the structural model in this analysis and an alternative transformation for compositional data (additive log-ratio (ALR)). Appendix A Table A1 includes two measures of goodness-of-fit, the Akaike Information Criterion and the Bayesian Information Criterion. Both metrics demonstrate strong evidence of better model fitting using ILR transformation.

**Table A1:** Goodness-of-fit statistics for structural model compared to alternative.

Model	Akaike Information Criterion	Bayesian Information Criterion
Isometric Log-Ratio Transformation	−109.132	−76.072
Additive Log-Ratio Transformation	−80.086	−46.903

Appendix A Table A2 displays regression coefficients, variances, and indirect and total effects for the alternative (ALR) transformation for the structural model. The direct, indirect, and total effects of this model are largely similar to their corresponding coefficients in the primary model, demonstrating a larger total effect for DO compared to MD and MD compared to ZD.

**Table A2:** Alternative structural model results: regression coefficients, variances, indirect effects, and total effects.

Parameter	Outcome (Y)	Explanatory (X)	Estimate	Standard Error	*p*-Value
Regressions					
$\beta_{1}$	DTP3	DO	−0.131	0.005	0.000
$\beta_{2}$	DTP3	MD	−0.051	0.004	0.000
$\beta_{3}$	DTP3	ZD	−0.036	0.002	0.000
$\alpha_{1}$	DO	ZD	0.104	0.021	0.000
$\alpha_{2}$	DO	MD	0.522	0.033	0.000
$\gamma_{1}$	MD	ZD	0.320	0.032	0.000
Variances					
	DTP3		0.001	0.000	0.000
	DO		0.174	0.014	0.000
	MD		0.557	0.046	0.000
Indirect Effects					
$\alpha_{1}\times \beta_{1}$	DTP3 (indirect effect via DO)	ZD	−0.014	0.003	0.000
$\gamma_{1}\times \beta_{2}$	DTP3 (indirect effect via MD)	ZD	−0.016	0.002	0.000
$(\alpha_{1}\times \beta_{1})+\left(\gamma_{1}\times \beta_{2}\right)$	DTP3 (total indirect effect)	ZD	−0.030	0.003	0.000
$\alpha_{2}\times \beta_{1}$	DTP3 (total indirect effect)	MD	−0.068	0.005	0.000
Total Effects					
$(\alpha_{1}\times \beta_{1})+\left(\gamma_{1}\times \beta_{2}\right)+\beta_{3}\;$	DTP3 (total effect)	ZD	−0.066	0.004	0.000
$\left(\alpha_{2}\times \beta_{1}\right)+\beta_{2}\;$	DTP3 (total effect)	MD	−0.119	0.004	0.000
$\beta_{1}$	DTP3	DO	−0.131	0.008	0.000
