# Supplementary material for: Variations in Routine Childhood Vaccination Gaps: A Decomposition Analysis Across 80 Low- and Middle-Income Countries
Source: Vaccines (Basel). 2025 Nov 4;13(11):1136. doi: 10.3390/vaccines13111136 (PMC12656951; doi:10.3390/vaccines13111136)
Supplement: Supplementary file 1 [file vaccines-13-01136-s001.zip › vaccines-3926387-supplementary.pdf]

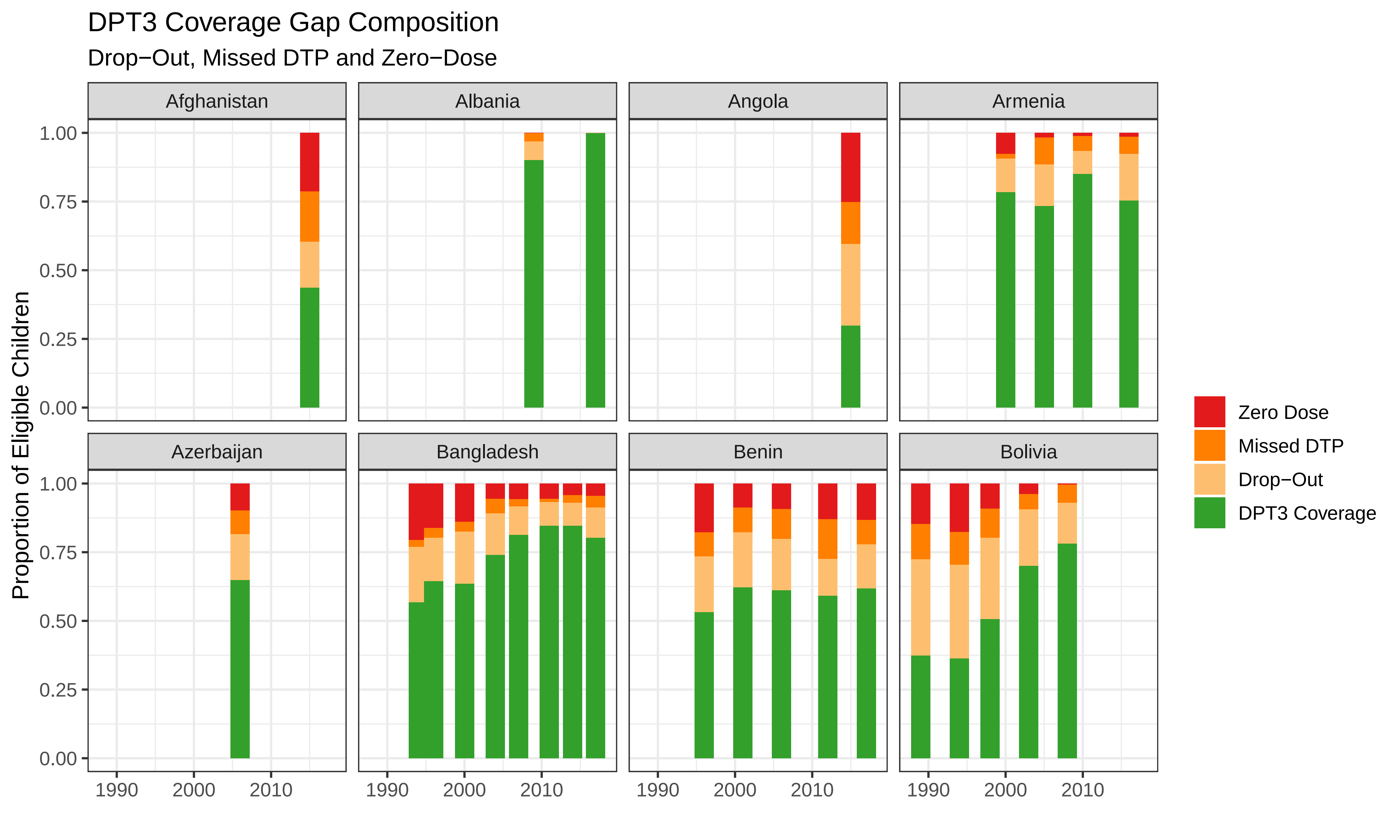

DPT3 Coverage Gap Composition
Drop−Out, Missed DTP and Zero−Dose
Afghanistan
Albania
Angola
Armenia
Azerbaijan
Bangladesh
Benin
Bolivia
Proportion of Eligible Children
0.00
0.25
0.50
0.75
1.00
1990
2000
2010
Zero Dose
Missed DTP
Drop−Out
DPT3 Coverage

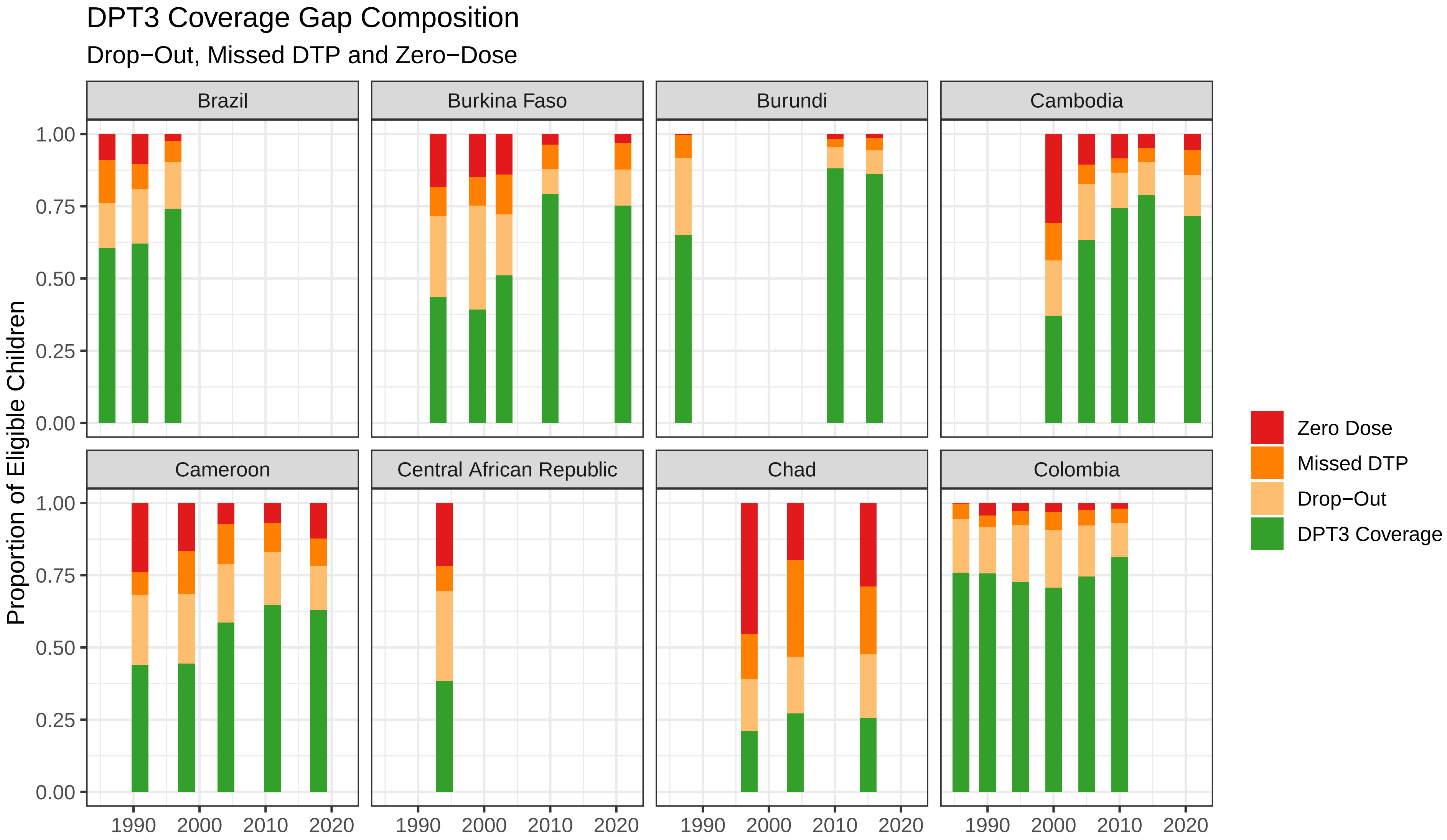

DPT3 Coverage Gap Composition
Drop−Out, Missed DTP and Zero−Dose
Brazil
Burkina Faso
Burundi
Cambodia
Cameroon
Central African Republic
Chad
Colombia
Proportion of Eligible Children
0.00
0.25
0.50
0.75
1.00
1990
2000
2010
2020
Zero Dose
Missed DTP
Drop−Out
DPT3 Coverage

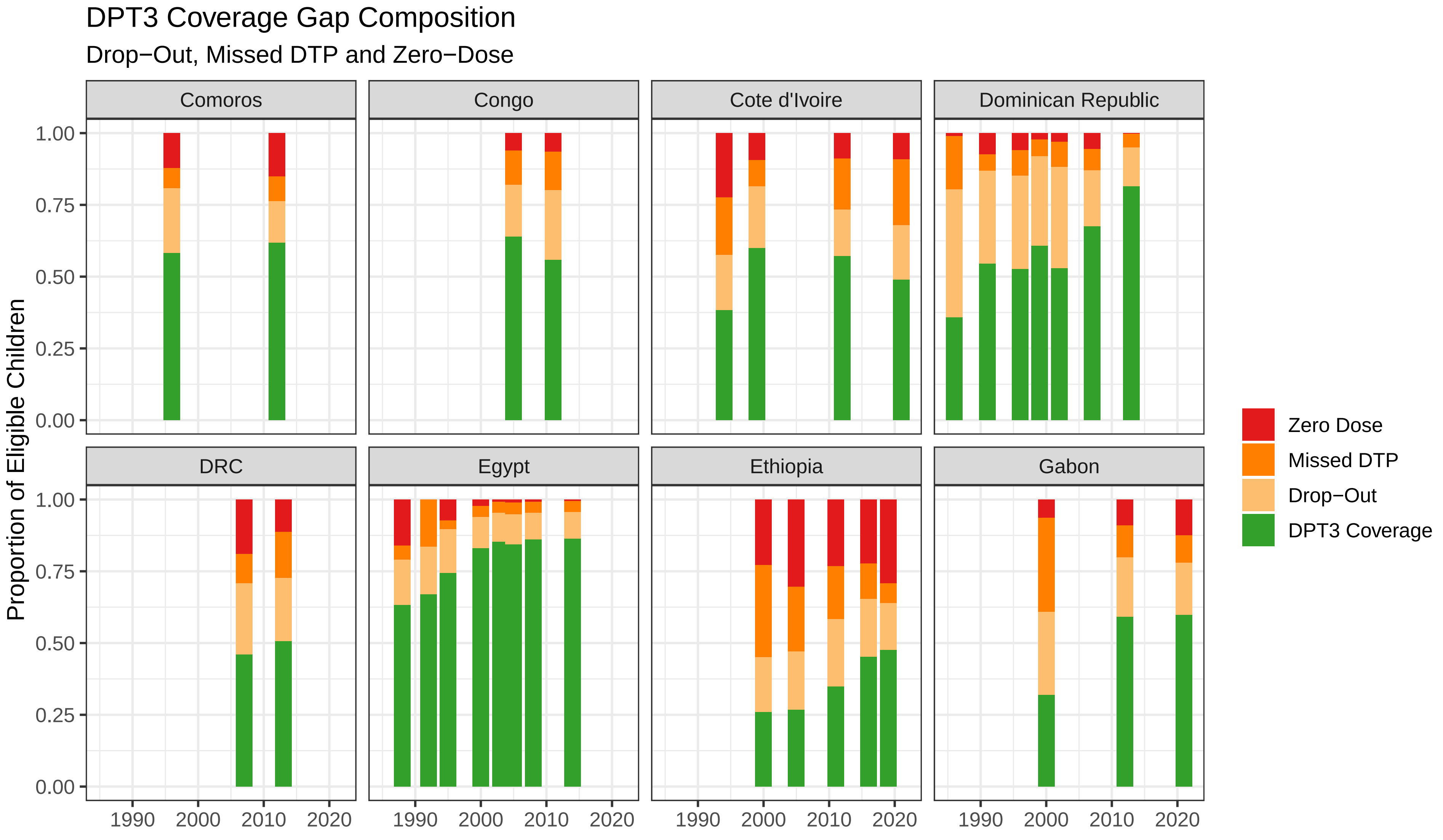
DPT3 Coverage Gap Composition
Drop−Out, Missed DTP and Zero−Dose
Comoros
Congo
Cote d'Ivoire
Dominican Republic
DRC
Egypt
Ethiopia
Gabon
Proportion of Eligible Children
0.00
0.25
0.50
0.75
1.00
1990
2000
2010
2020
Zero Dose
Missed DTP
Drop−Out
DPT3 Coverage

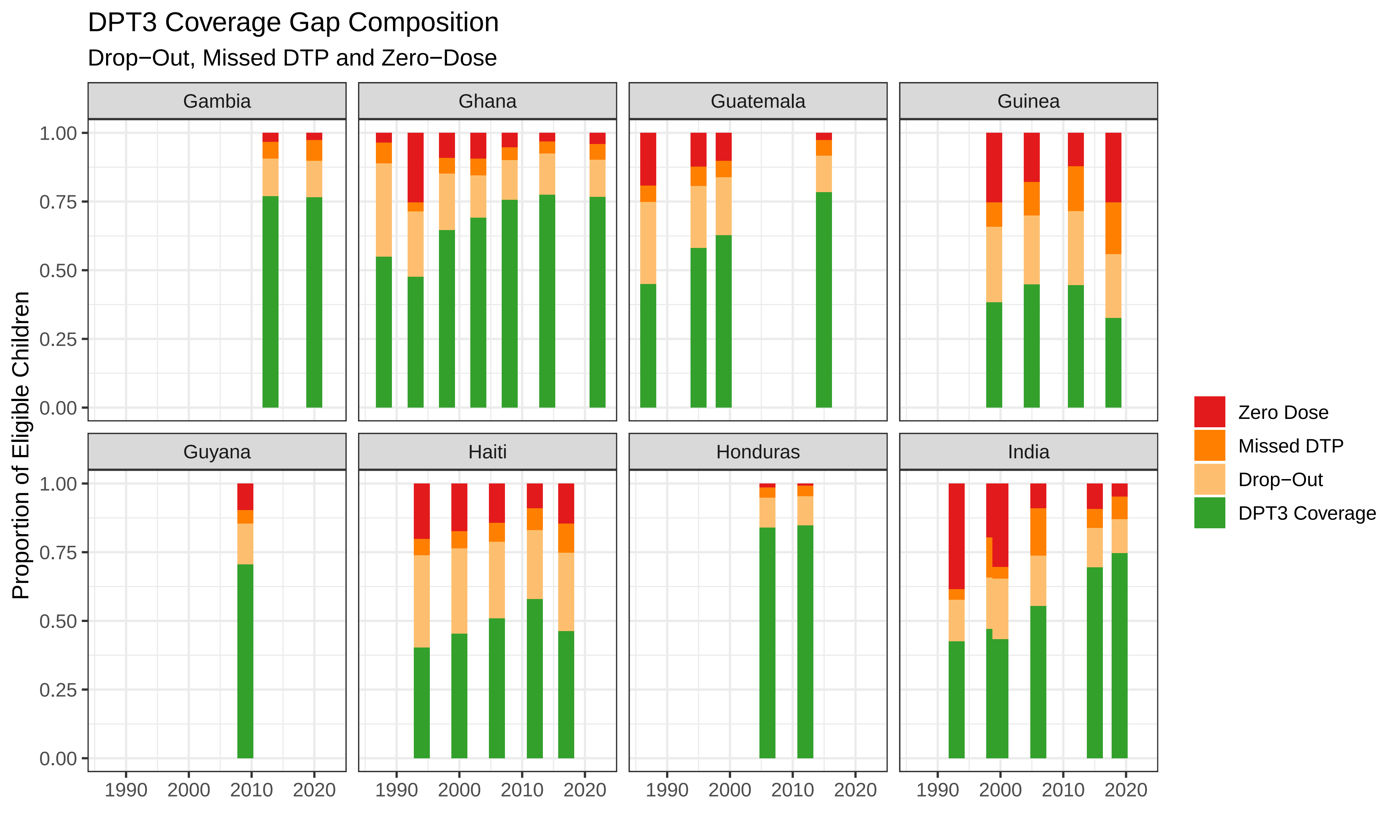

DPT3 Coverage Gap Composition
Drop−Out, Missed DTP and Zero−Dose
Gambia
Ghana
Guatemala
Guinea
Guyana
Haiti
Honduras
India
Proportion of Eligible Children
1.00
0.75
0.50
0.25
0.00
1990
2000
2010
2020
Zero Dose
Missed DTP
Drop−Out
DPT3 Coverage

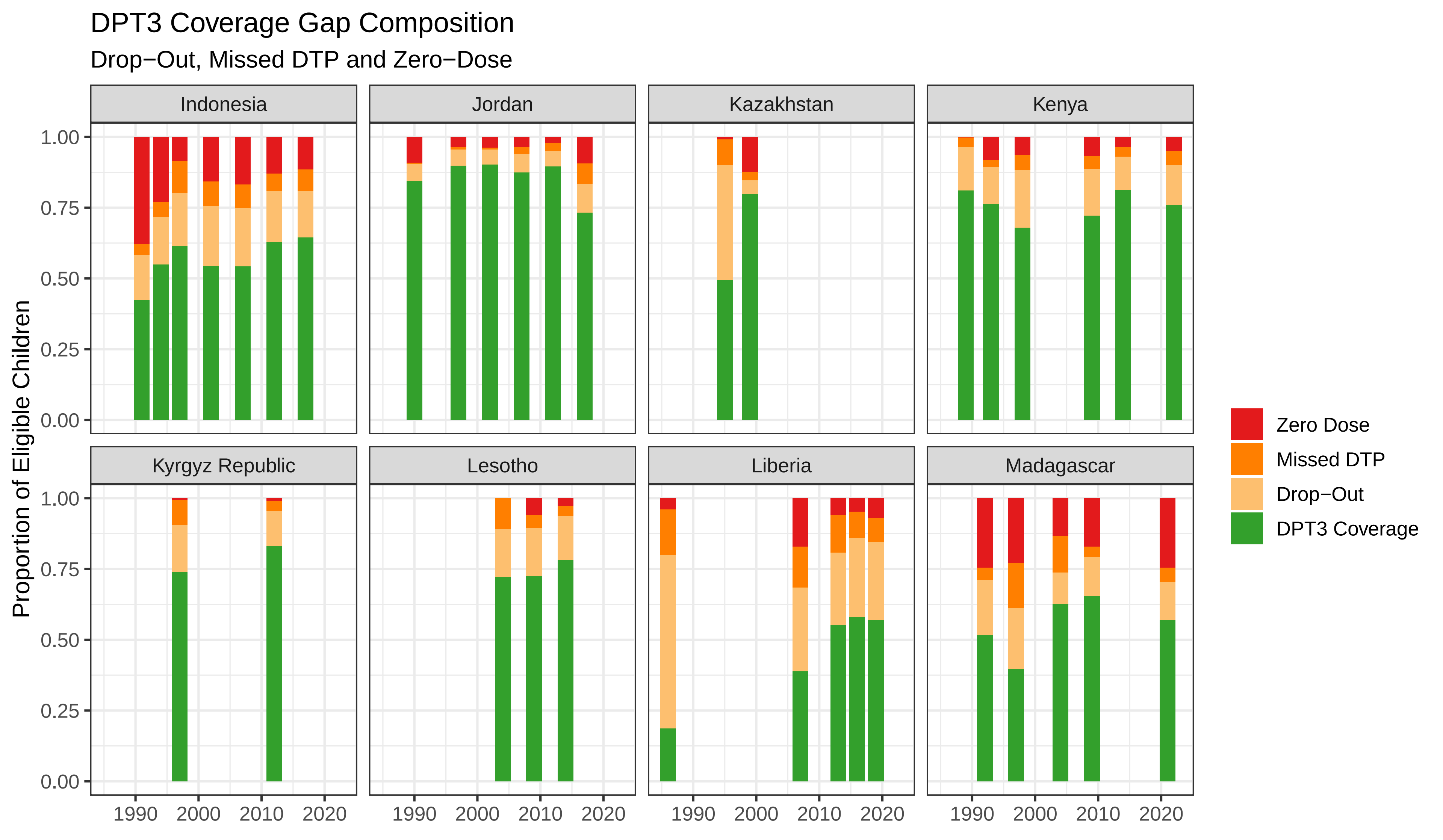
DPT3 Coverage Gap Composition
Drop−Out, Missed DTP and Zero−Dose
Proportion of Eligible Children
Indonesia
Jordan
Kazakhstan
Kenya
Kyrgyz Republic
Lesotho
Liberia
Madagascar
0.00
0.25
0.50
0.75
1.00
1990
2000
2010
2020
Zero Dose
Missed DTP
Drop−Out
DPT3 Coverage

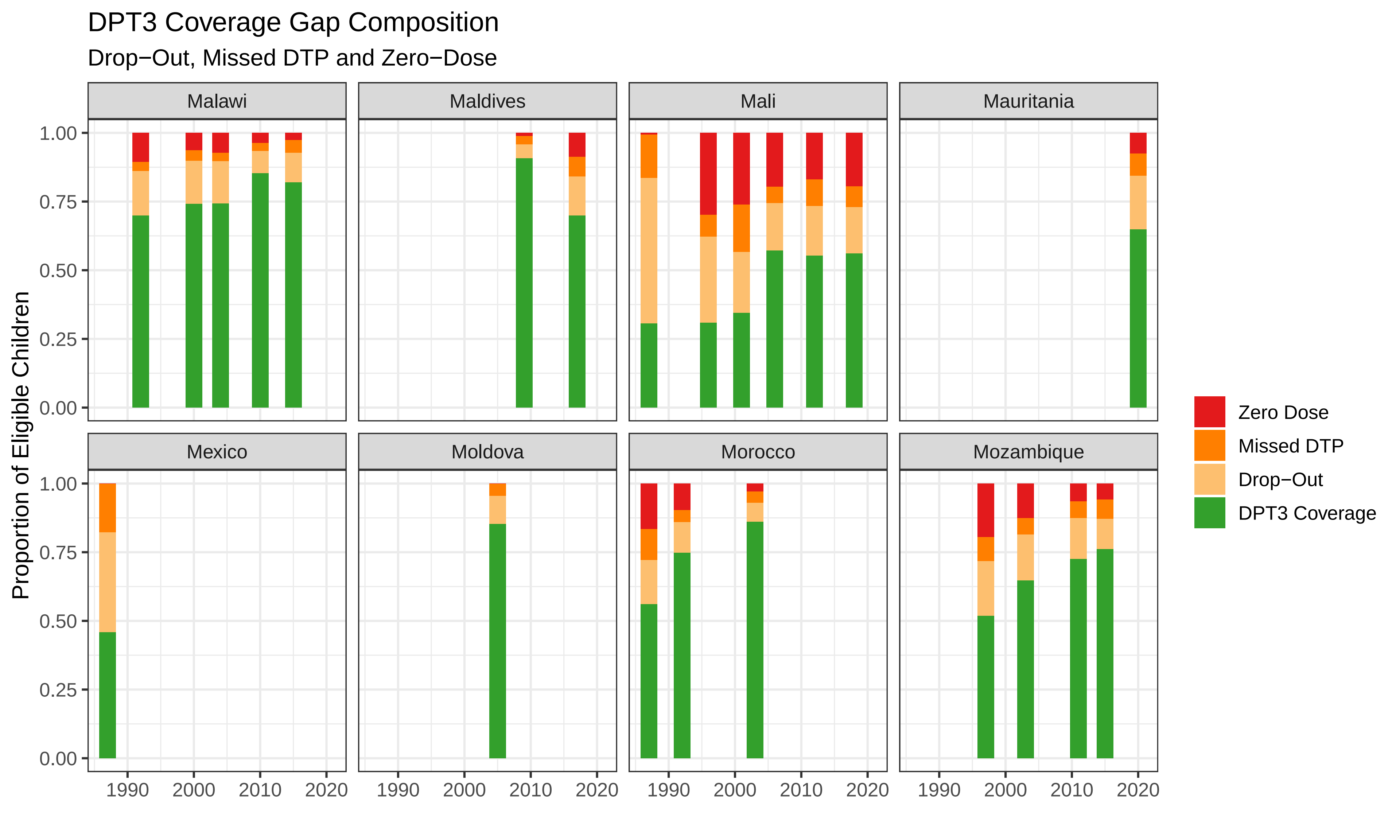

DPT3 Coverage Gap Composition
Drop−Out, Missed DTP and Zero−Dose
Malawi
Maldives
Mali
Mauritania
Mexico
Moldova
Morocco
Mozambique
Proportion of Eligible Children
0.00
0.25
0.50
0.75
1.00
1990
2000
2010
2020
Zero Dose
Missed DTP
Drop−Out
DPT3 Coverage

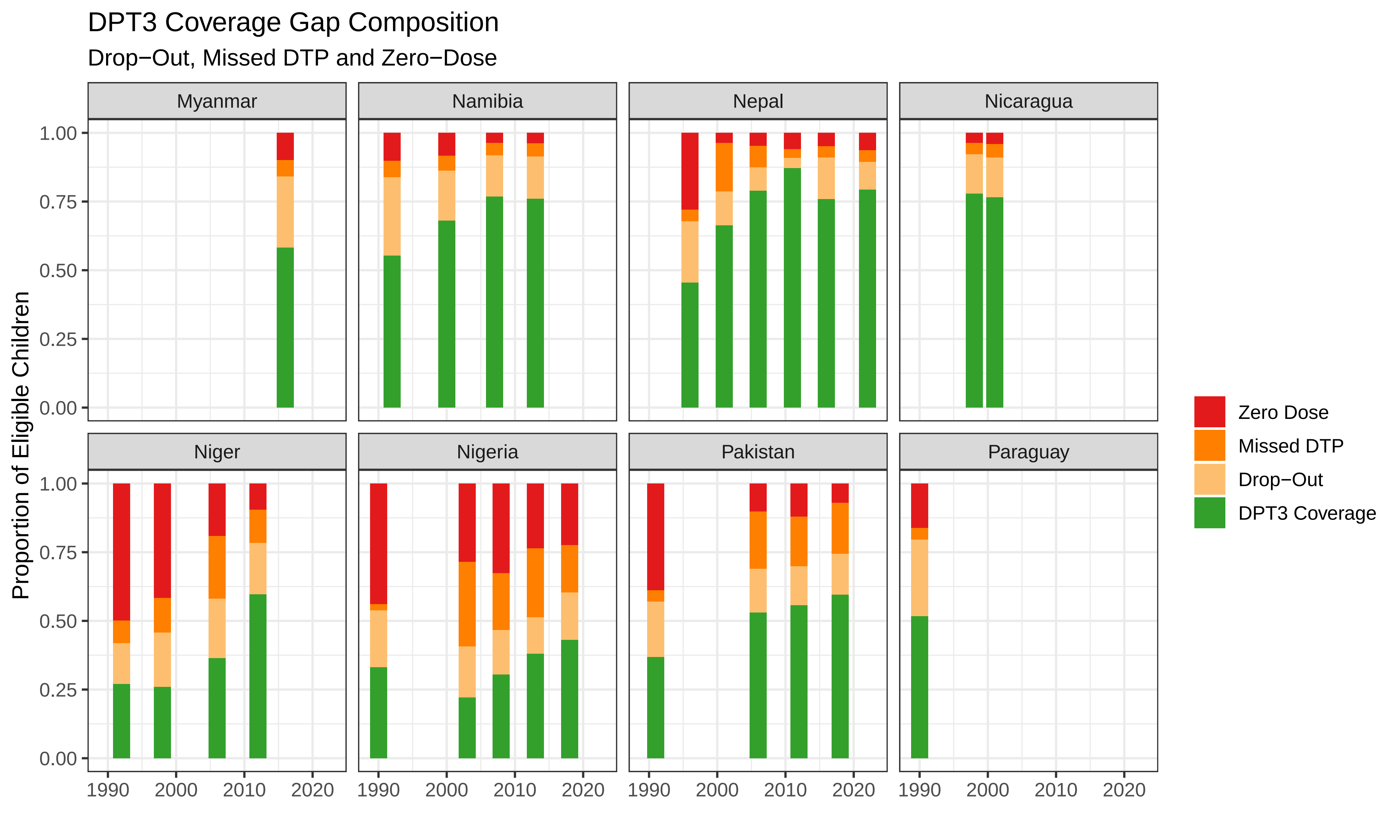

DPT3 Coverage Gap Composition
Drop−Out, Missed DTP and Zero−Dose
Myanmar
Namibia
Nepal
Nicaragua
Niger
Nigeria
Pakistan
Paraguay
Proportion of Eligible Children
0.00
0.25
0.50
0.75
1.00
1990
2000
2010
2020
Zero Dose
Missed DTP
Drop−Out
DPT3 Coverage

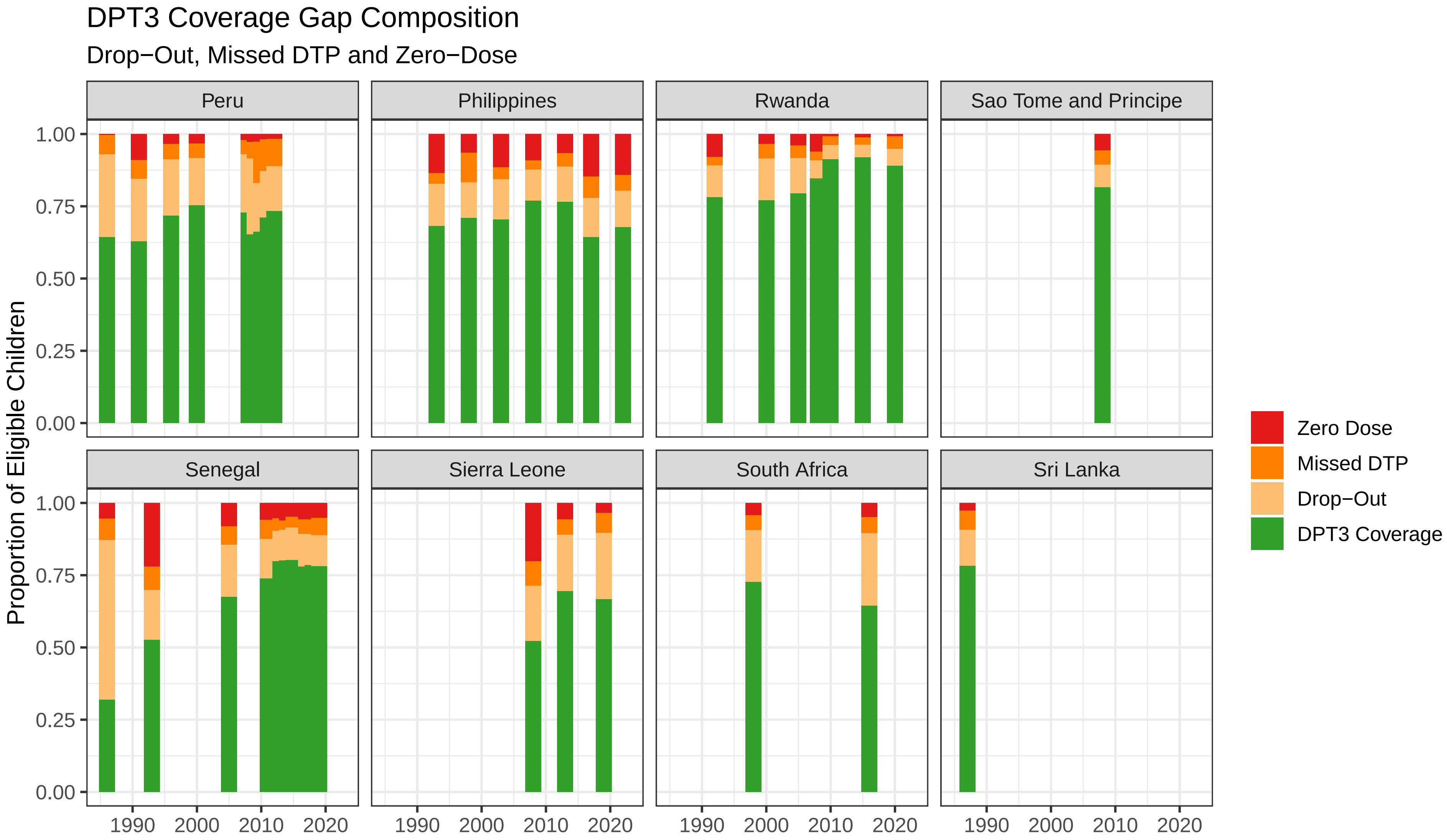

DPT3 Coverage Gap Composition
Drop−Out, Missed DTP and Zero−Dose
Peru
Philippines
Rwanda
Sao Tome and Principe
Senegal
Sierra Leone
South Africa
Sri Lanka
Proportion of Eligible Children
0.00
0.25
0.50
0.75
1.00
1990
2000
2010
2020
Zero Dose
Missed DTP
Drop−Out
DPT3 Coverage

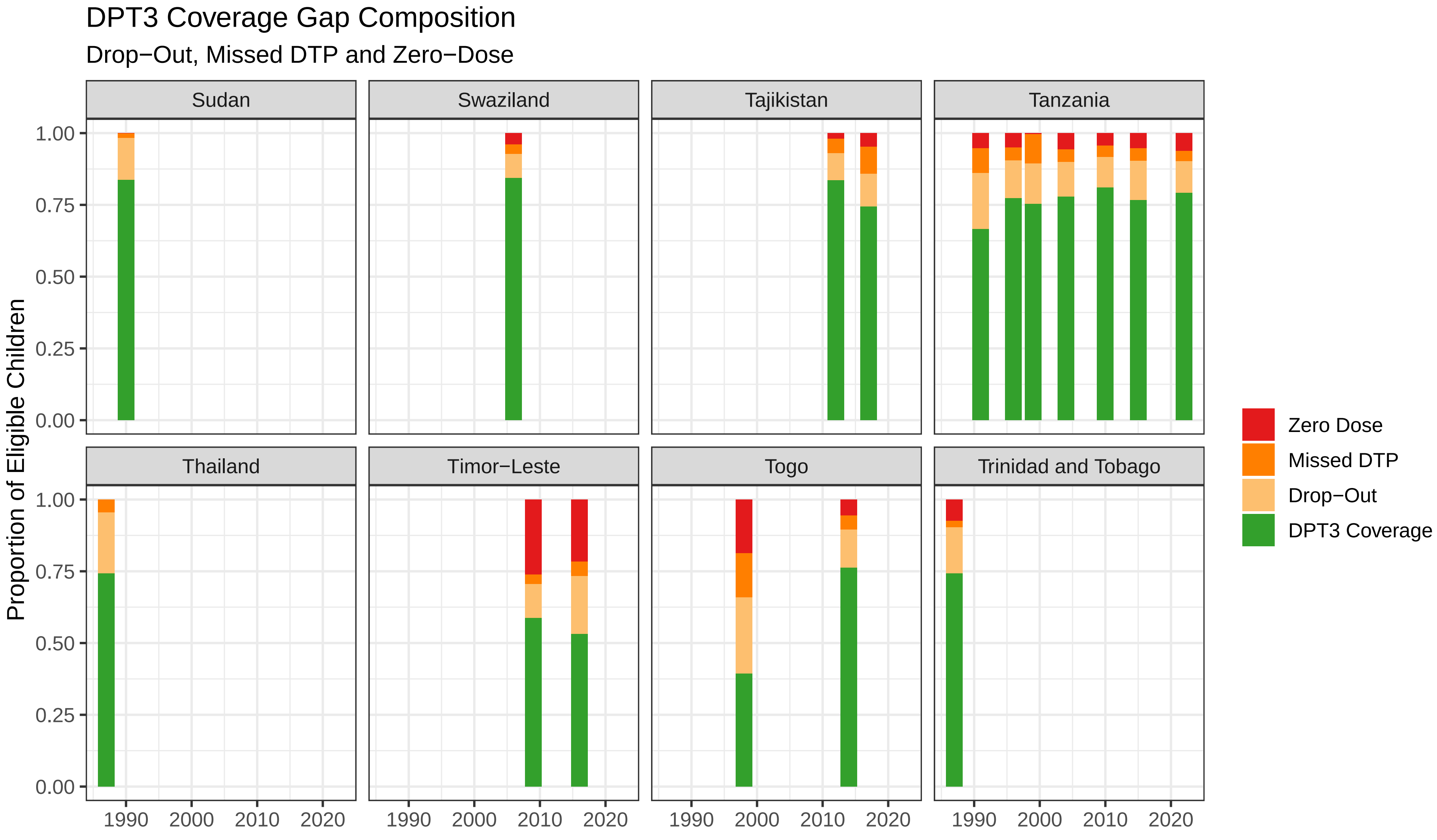

DPT3 Coverage Gap Composition
Drop−Out, Missed DTP and Zero−Dose
Sudan
Swaziland
Tajikistan
Tanzania
Thailand
Timor−Leste
Togo
Trinidad and Tobago
Proportion of Eligible Children
0.00
0.25
0.50
0.75
1.00
1990
2000
2010
2020
Zero Dose
Missed DTP
Drop−Out
DPT3 Coverage

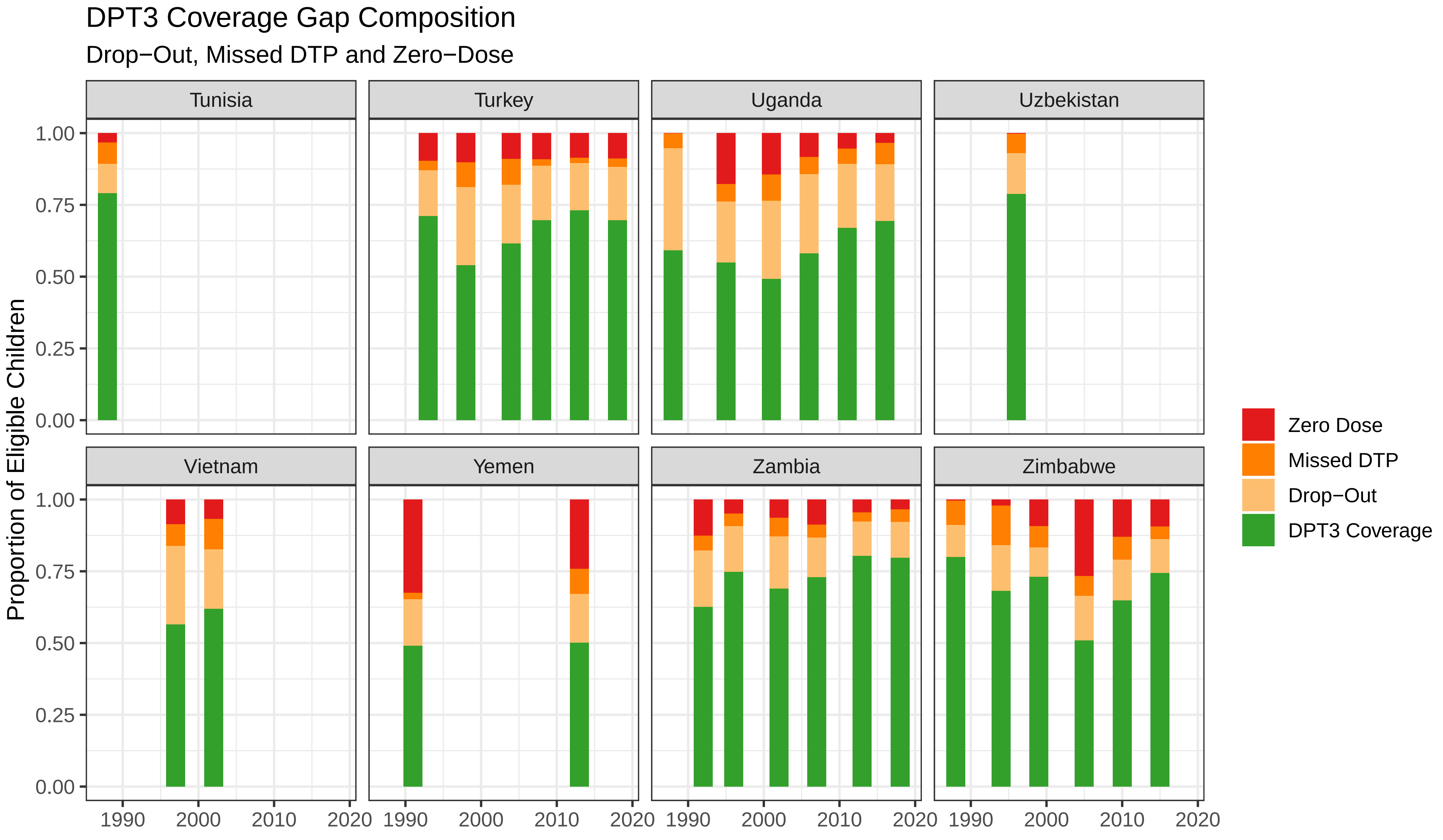

DPT3 Coverage Gap Composition
Drop−Out, Missed DTP and Zero−Dose
Tunisia
Turkey
Uganda
Uzbekistan
Vietnam
Yemen
Zambia
Zimbabwe
Proportion of Eligible Children
0.00
0.25
0.50
0.75
1.00
1990
2000
2010
2020
Zero Dose
Missed DTP
Drop−Out
DPT3 Coverage
